# 基于超高效液相色谱-串联质谱的股骨头坏死组织外泌体脂质代谢组学分析

**DOI:** 10.3724/SP.J.1123.2021.04016

**Published:** 2022-02-08

**Authors:** Minkang GUO, Jian ZHANG

**Affiliations:** 重庆医科大学附属第一医院骨科, 重庆 400016; Department of Orthopedics, the First Affiliated Hospital of Chongqing Medical University, Chongqing 400016, China; 重庆医科大学附属第一医院骨科, 重庆 400016; Department of Orthopedics, the First Affiliated Hospital of Chongqing Medical University, Chongqing 400016, China

**Keywords:** 超高效液相色谱-串联质谱, 代谢组学, 代谢通路, 股骨头坏死, 外泌体, 脂质, ultra performance liquid chromatography-tandem mass spectrometry (UPLC-MS/MS), metabolomics, metabolic pathway, osteonecrosis of the femoral head (ONFH), exosomes, lipid

## Abstract

股骨头坏死(ONFH)是一种可导致股骨头塌陷进而需要接受全髋关节置换的疾病。外泌体作为一种细胞间交流的方式,在一系列生理和病理过程中起着至关重要的作用,已在疾病的诊断和治疗中发挥独特作用。该研究利用非靶向代谢组学方法,探讨股骨头坏死组织外泌体内的脂质代谢特征,阐释股骨头坏死时机体发生的脂质代谢变化。该研究采用超速离心的方法,对股骨头坏死组织的外泌体进行了分离富集,并使用动态光散射(DLS)、蛋白质免疫印迹和透射电子显微镜(TEM)3种方法鉴定外泌体。采用超高效液相色谱-串联质谱(UPLC-MS/MS)结合多变量统计分析识别股骨头坏死外泌体的脂质代谢谱。采用主成分分析(PCA)和正交偏最小二乘判别分析(OPLS-DA)对差异表达的外泌体脂质代谢物进行多变量统计分析。在外泌体中检测到18种明显改变的脂质代谢物,包括丙烯醇酯类、脂肪酸酯类、甘油酯类及其衍生物。通过代谢分析网站进行通路分析,从而确定受影响的代谢通路并进行可视化。代谢通路分析显示外泌体内的甘油磷脂代谢和鞘脂代谢改变最为明显,鞘脂和甘油磷脂之间的不平衡导致脂肪毒性损伤,这与常见代谢性疾病的病理生理学有关。同时,甘油磷脂与细胞增殖、分化和凋亡之间具有相关性,甘油磷脂比例的变化可以反映脂质代谢的紊乱。外泌体内的脂质代谢变化可能在一定程度上反映了ONFH疾病的代谢变化。ONFH外泌体脂质代谢组学分析可有助于探索坏死骨组织外泌体中的脂质代谢变化和受影响的脂质代谢通路。

## Abstract (en)

Osteonecrosis of the femoral head (ONFH) can lead to its collapse which requires total hip arthroplasty. Exosomes, which are important for intercellular communication are involved in a series of physiological and pathological processes, and therefore play a unique role in disease diagnosis and treatment. In this study, untargeted metabolomics was used to investigate the metabolic characteristics of lipids in exosomes of femoral head tissue with osteonecrosis and to explain the metabolic changes that occur in the body during this disease. Ultracentrifugation was used to separate and enrich exosomes from femoral head tissue with osteonecrosis. Exosomes were identified using dynamic light scattering (DLS), Western blotting, and transmission electron microscopy (TEM). Gradient elution was performed with ultrapure water and acetonitrile as mobile phases using a Kinetex XB-C18 column (100 mm×2.1 mm, 2.6 μm). The column oven temperature, flow rate of the mobile phase, and duration were 30 ℃, 300 μL/min, and 15 min, respectively. A triple TOF 4600 high resolution mass spectrometry system was used, and the mass scan range of *m/z* was set at 100 -1000. Other conditions were as follows: sheath gas, 380 kPa; auxiliary gas, 380 kPa; curtain gas, 170 kPa; and atomization temperature, 600 ℃. Ultra performance liquid chromatography-tandem mass spectrometry (UPLC-MS/MS) combined with multivariate statistical analysis was used to identify the lipid metabolic profile of ONFH-derived exosomes. The exosome metabolites were characterized in detail, which enables their identification and provided a reliable method for quality evaluation. After transforming the obtained original data using MarkView software, peak identification, peak alignment, subtraction of solvent peak, impurity peak, noise filtering, and other treatments, a three-dimensional matrix was obtained from the exported data table. Principal component analysis (PCA) and orthogonal partial least squares discrimination analysis (OPLS-DA) in the SIMCA-P14.1 software were used for multivariate statistical analysis of differentially expressed exosome lipid metabolites. This strategy was validated using lipid metabolites from patients with ONFH and healthy controls. The correlation distribution was shown according to the point dispersion of the PCA score plot, and lipid metabolites from the same disease showed ideal clustering. This result indicates a small difference between the groups. A good clustering effect is also obtained using OPLS-DA, and the statistical model has high reliability. A total of 18 significantly altered lipid metabolites were detected in the exosomes, including acrylolipids, fatty acid esters, glycerides, and their derivatives. The pathway analysis was conducted with MetaboAnalyst (https://www.metaboanalyst.ca/) via database source including the HMDB (http://www.hmdb.ca/) and MMCD (http://mmcd.nmrfam.wisc.edu/) for confirming the impacted metabolic pathways and visualization. Metabolic pathway analysis showed that glycerophospholipid and sphingolipid metabolism were the most significantly altered in exosomes. An imbalance between sphingolipids and glycerophospholipids leads to lipotoxic damage, which is implicated in the pathophysiology of common metabolic diseases. Furthermore, glycerophospholipids are correlated with cell proliferation, differentiation, and apoptosis, and the change in glycerophospholipid ratio can reflect the disturbance in lipid metabolism. The metabolic changes in exosomes may reflect the metabolic changes in ONFH. In this study, lipid metabolomics analysis based on UPLC-MS/MS was used to determine metabolic differences between exosomes extracted from ONFN and femoral neck fracture (FNF). Metabolomic analysis of necrotic femoral head tissue-derived exosomes can help explore the most relevant pathways for assessing the changes in exosome metabolism that affect exosome metabolism in necrotic bone tissue.

股骨头坏死(ONFH)是一种可导致股骨头塌陷进而需要接受全髋关节置换的疾病。该病严重影响患者的日常生活,给社会造成了重大的经济负担,但其发生机制和病理生理仍不清楚^[1,2]^。Bartolmas等^[3]^证实甘油磷脂在调节生理病理过程中发挥着重要作用。溶血磷脂酸(LPA)和鞘氨醇-1-磷酸(S1P)可促进内皮细胞迁移和血管生成。在小鼠去卵巢骨质疏松模型中,脂质代谢紊乱与骨吸收和骨形成的失衡密切相关^[4]^。同时,许多研究认为脂质代谢紊乱为ONFH的代谢特征之一^[5,6,7]^。

外泌体是一种直径为30~150 nm的细胞外囊泡,被双层脂膜包裹,其分泌由细胞高度调控^[8]^。外泌体作为一种细胞间交流的方式,在一系列生理和病理过程中起着至关重要的作用。外泌体可以在受体细胞中产生生物反应,并转运小分子代谢物^[9]^。据报道,骨髓来源的间充质干细胞分泌的外泌体参与血管生成,调节骨代谢^[10]^。前期研究表明,来自正常股骨头组织的外泌体可减少糖皮质激素诱导的大鼠模型ONFH的发生,促进间充质干细胞的成骨分化和迁移;基于蛋白质组学分析,血小板表面糖蛋白(CD41)缺陷的外泌体可导致ONFH的发生,但来自正常骨骼的外泌体可以改善ONFH的进展^[11]^,然而其机制尚不清楚,因为外泌体包含代谢产物等分子。因此,进一步研究来自ONFH骨组织外泌体中代谢物的作用是必要的,这可能为外泌体介导的代谢途径提供思路,加深我们对ONFH的理解。

代谢组学是对某一生物或细胞在一特定生理时期内所有相对分子质量较低代谢产物同时进行定性和定量分析的一门新学科^[12]^。代谢物是细胞功能的组成部分,被认为拥有大量能预测表型的信息^[13]^。超高效液相色谱-串联质谱(UPLC-MS/MS)技术以其高精度和高灵敏度被广泛用于组学分析^[14,15]^。本研究基于UPLC-MS/MS的非靶向代谢组学技术分析了来源于ONFH骨组织外泌体内脂质代谢物的变化。本研究旨在为观察ONFH提供一个新的视角,并探索ONFH疾病外泌体中可能的代谢变化。

## 1 实验部分

### 1.1 仪器、试剂与材料

高效液相色谱系统(UltiMate 3000)、超速离心机(Sorvall WX)(美国Thermo Fisher公司);透射电子显微镜(H-7650,日本Hitachi公司);动态光散射仪(LS-13,美国Berkam公司); Kinetex XB-C18色谱柱(100 mm×2.1 mm, 2.6 μm, 美国Phenomenex公司); Triple TOF 5600 LC-MS系统(美国SCIEX公司); Milli-Q超纯水系统(美国Millipore公司)。

RIPA裂解液和BCA蛋白浓度检测试剂盒均购自上海碧云天生物技术有限公司;乙腈和甲醇(色谱级,美国Thermo Fisher公司);甲酸(色谱级,德国CNW公司);聚偏氟乙烯膜(美国Millipore Corporation公司);洗膜缓冲液(TBST)、磷酸缓冲盐溶液(PBS, 0.01 mol/L, PH 7.4)和5%牛血清白蛋白封闭液(BSA)均购自武汉博士德生物工程有限公司;第一抗体:Alix-1和CD63均购自沈阳万类生物科技有限公司,TSG101购自美国Abcam公司;第二抗体:辣根过氧化物酶(HRP)标记山羊抗兔IgG (上海碧云天生物技术有限公司)。

### 1.2 样本收集

所有实验均通过重庆医科大学附属第一医院伦理委员会的批准。本研究中的捐赠者均充分了解知情同意书的内容,同时也获得了每位捐赠者的书面知情同意。ONFH患者的纳入标准:(1)X射线、核磁共振(MRI)及病理表现与ONFH诊断一致;(2)根据国际骨循环研究会(ARCO)分期,患者处于Ⅲ~Ⅳ期;(3)患者未出现严重内源性疾病、畸形性骨炎、代谢性骨病、转移性骨癌、甲状旁腺功能亢进等疾病。本研究涉及30例ONFH患者及30例股骨颈骨折(FNF)患者的标本。患者的人口学统计数据见表1。

**表 1:** 入组样本的临床信息(*n*=30)

Character	Age/year	Gender(male/female)	Height/cm	Weight/kg	BMI/(kg/m^2^)
FNF	72.28±8.27	12/18	156.47±5.68	56.16±8.44	22.03±2.51
ONFH	65.57±5.14	17/13	163.43±5.54	61.78±7.94	23.62±3.27
P	0.063	0.196	0.035	0.036	0.063

Data are presented as mean±SD. BMI: body mass index; FNF: femoral neck fracture; ONFH: osteonecrosis of the femoral head. Differences were considered significant at *P*<0.05.

### 1.3 超速离心法提取外泌体

外泌体的分离纯化采用多级超速离心法进行。将500 mg股骨头组织充分研磨后与PBS混合,于4 ℃条件下以300 g离心10 min,随后将上清液吸至另一高速离心管中,于4 ℃下以10000 g离心30 min,最后取上清液,于4 ℃下以100000 g离心70 min,获得外泌体沉淀,使用PBS对外泌体进行重悬洗涤,以100000 g离心70 min,获得纯净的外泌体沉淀。最后,用PBS吹打外泌体,用0.22 μm滤器过滤,外泌体溶液的含量为1.9×10^11^ particles/mL,保存于-80 ℃。

### 1.4 蛋白免疫印迹分析

向外泌体样本中加入RIPA裂解液,在冰上裂解20 min后,以13000 r/min离心30 min提取总蛋白质。BCA试剂盒测定总蛋白质浓度后进行蛋白免疫印迹分析,蛋白质上样量为每孔30 μg。

十二烷基硫酸钠-聚丙烯酰胺凝胶电泳(SDS-PAGE)分离程序:以80 V电压运行30 min进行浓缩,以120 V电压运行60 min进行分离。转膜程序:以210 mA电流运行90 min,将蛋白质转移至聚偏氟乙烯膜(PVDF)上,在5% BSA中室温摇床封闭1 h。将封闭好的PVDF膜先在双蒸水中清洗掉封闭液,然后放入洗膜缓冲液(TBST)中,摇床每次清洗7 min,共3次。PVDF膜在4 ℃冰箱中与第一抗体孵育12 h后,PVDF膜放入TBST,摇床每次清洗7 min,共3次。最后,PVDF膜与第二抗体孵化1 h。

### 1.5 代谢物的提取

外泌体按照之前的方案制备样品^[16]^。具体实验步骤:以100 Hz超声持续5 min破碎外泌体,然后加入100 μL乙腈,于4 ℃下以12000 r/min离心10 min以去除微粒。取40 μL上清液,用60 μL蒸馏水稀释。将60 μL上清液装入玻璃自动进样瓶中。每一组的剩余上清取10 μL作为质控组。

### 1.6 分析条件

1.6.1 色谱条件

色谱柱:Kinetex XB-C18色谱柱(100 mm×2.1 mm, 2.6 μm);柱温:30 ℃;流动相:(A)0.1%(v/v)甲酸水溶液和(B)0.1%(v/v)甲酸乙腈溶液;流速:0.3 mL/min,样品室温度:8 ℃。梯度洗脱程序:0~1.0 min, 10%B; 1.0~8.0 min, 10%B~80%B; 8.0~12.0 min, 80%B; 12.0~15.0 min, 80%B~10%B。进样量:60 μL。

1.6.2 质谱条件

质量扫描范围*m/z*: 100~1000,鞘气压力为380 kPa,辅助气压力为380 kPa,气帘气压力为170 kPa,雾化温度为600 ℃,采用飞行时间质谱全扫描-信息关联采集-子离子扫描(TOF-MS scan-IDA-product ion scan)复合模式,TOF-MS一级预扫描和触发的二级扫描IDA离子累积时间分别为250、100 ms,采用多重质量亏损(MMDF)和动态背景扣除(DBS)作为二级触发条件,解簇电压为±80 V,碰撞能量叠加为(35±15) eV。

### 1.7 数据分析

将所得的原始数据通过MarkView软件转化后,进行峰识别、峰对齐、扣除溶剂峰、杂质峰、滤噪等处理,利用快捷峰视图(Shortcut to PeakView)技术,通过化学智能峰匹配算法促进碎片离子分析过程,使用MMCD (http://mmcd.nmrfam.wisc.edu/)和HMDB (http://www.hmdb.ca/)数据库进行检索,由导出的数据表得到三维矩阵。经预处理后的数据矩阵导入SIMCA-P 14.1,进行多变量统计分析。依据正交偏最小二乘法判别分析(OPLS-DA)模型中变量投影重要性值(VIP值>1)和独立样本*t*检验(*P*<0.05)筛选差异代谢物。

## 2 结果与讨论

### 2.1 对受试者进行图像和组织学分析

ONFH患者的X射线图像显示股骨头密度变化,负重区塌陷,关节间隙狭窄(见图1a)。MRI图像显示软骨下骨折,信号强度较低(见图1b)。股骨头的大体形状如图1c所示,ONFH标本可见局灶性坏死区均匀改变,脂肪增多。苏木精-伊红染色法(HE)染色显示,ONFH骨组织存在坏死、纤维化、骨髓腔紊乱以及骨吸收(见图1d)。以上结果提示,ONFH患者股骨头的组织病理发生了改变。

**图 1:**
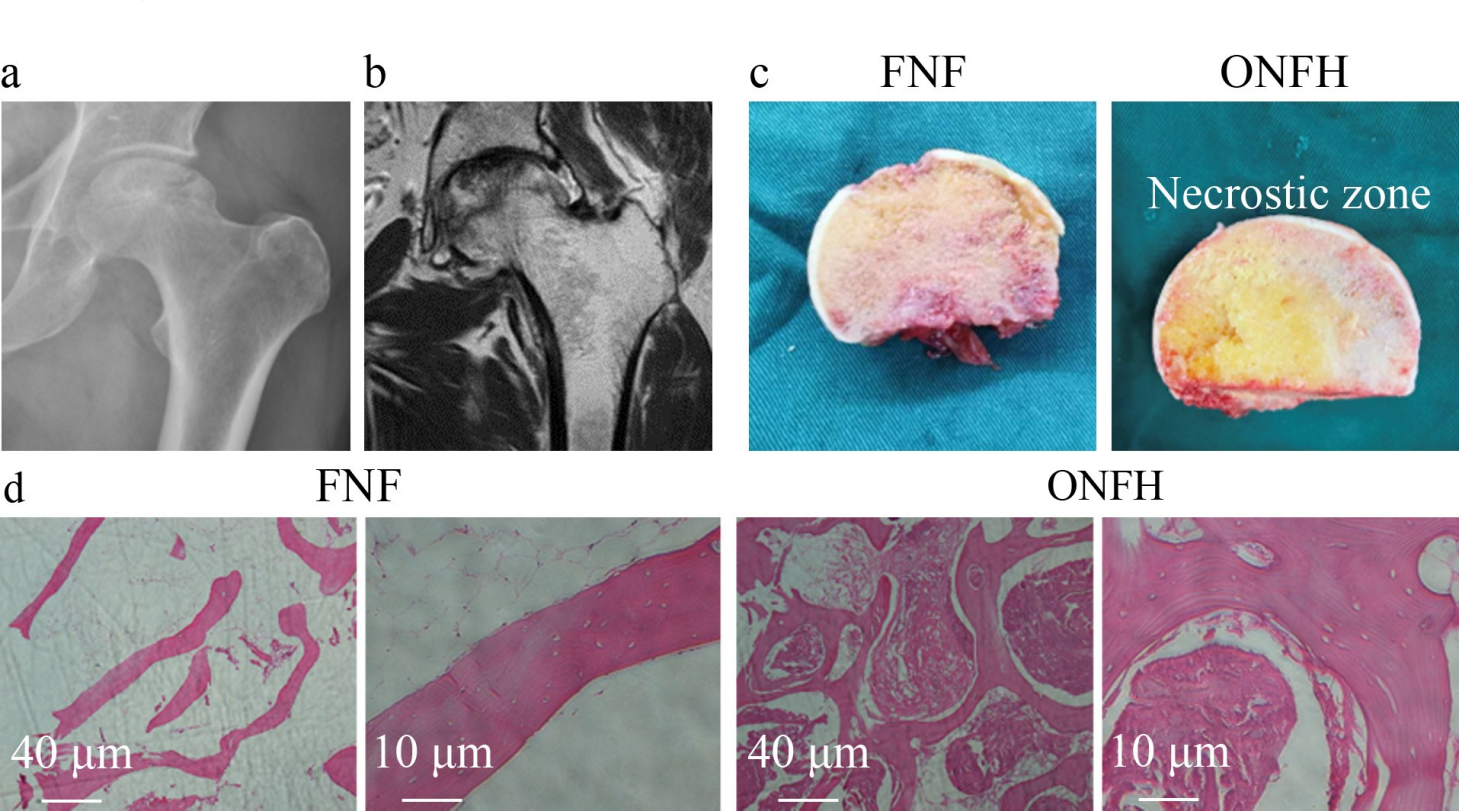
股骨头的组织图像和免疫组化图片

### 2.2 外泌体的鉴定

对分离富集得到的外泌体,分别进行动态光散射(DLS)分析、蛋白免疫印迹分析和透射电子显微镜(TEM)分析。DLS的结果显示,这些外泌体的大小大都在30~150 nm之间(见图2a)。图2b显示,外泌体的标记物CD63、Alix和TSG101显著富集。最后对外泌体进行了TEM分析,直接观察其形貌,结果如图2c所示,外泌体为双层脂质包裹,类圆盘状形态,大小在100 nm左右。以上结果表明组织中的外泌体已成功分离。

**图2:**
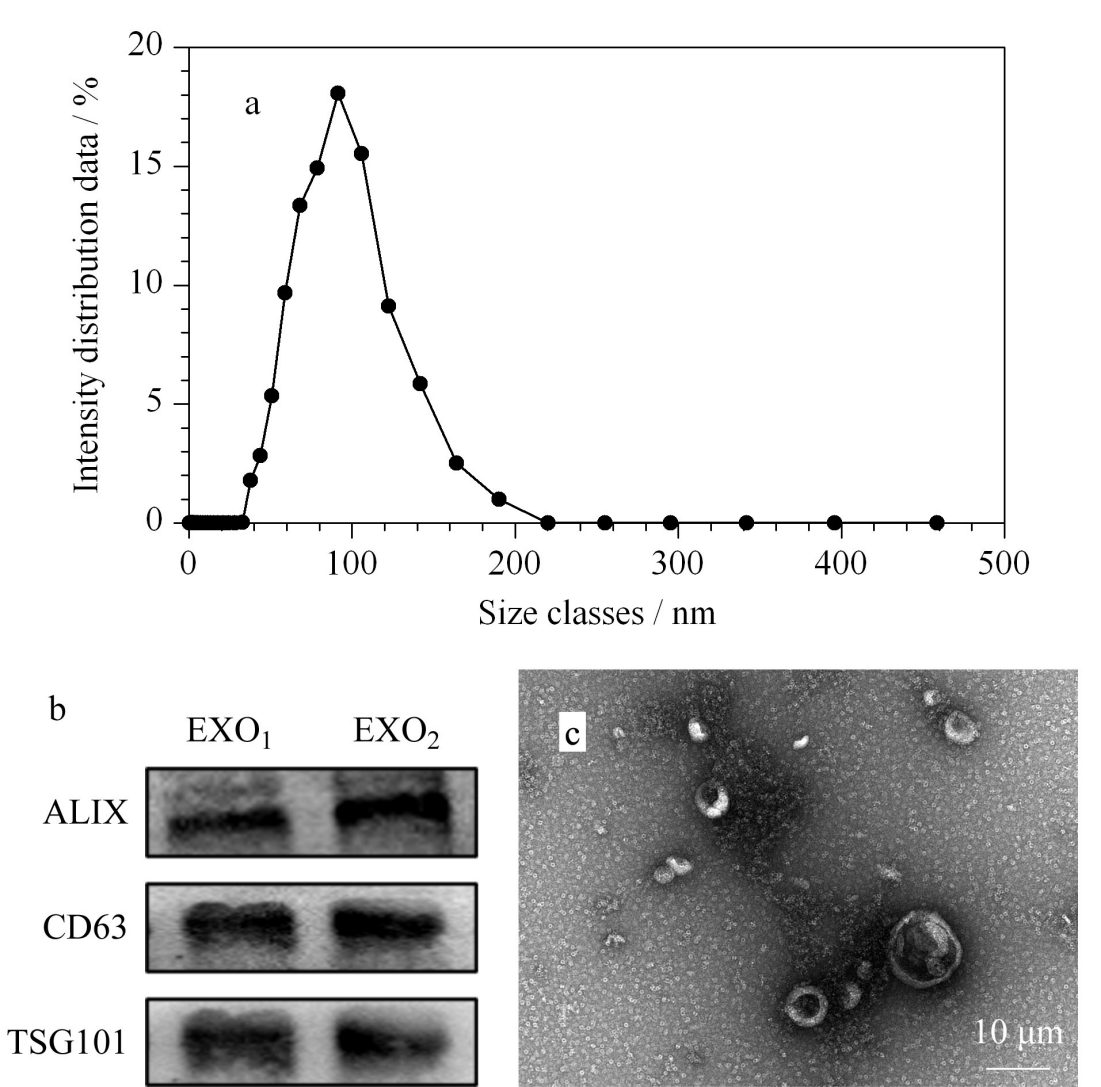
外泌体的鉴定

### 2.3 外泌体中脂质代谢产物谱

采用主成分分析(PCA)和正交偏最小二乘法判别分析(OPLS-DA)鉴别差异表达的代谢物。

外泌体样本经上述条件分析后得到的数据,经处理后得到PCA图(见图3a)。结果显示,来自于ONFH的外泌体的成分和来自于FNF的外泌体的成分在维度上,二者有一定的分离趋势,反映了ONFH的病理生理变化。通常情况下,*R*^2^*Y*、*Q*^2^高于0.5较好,高于0.4即可接受,且两者差值不应过大。临床样本由于个体差异大,不可控,尤其大样本时,*R*^2^*Y*、*Q*^2^大小为0.2左右亦可。

**图3:**
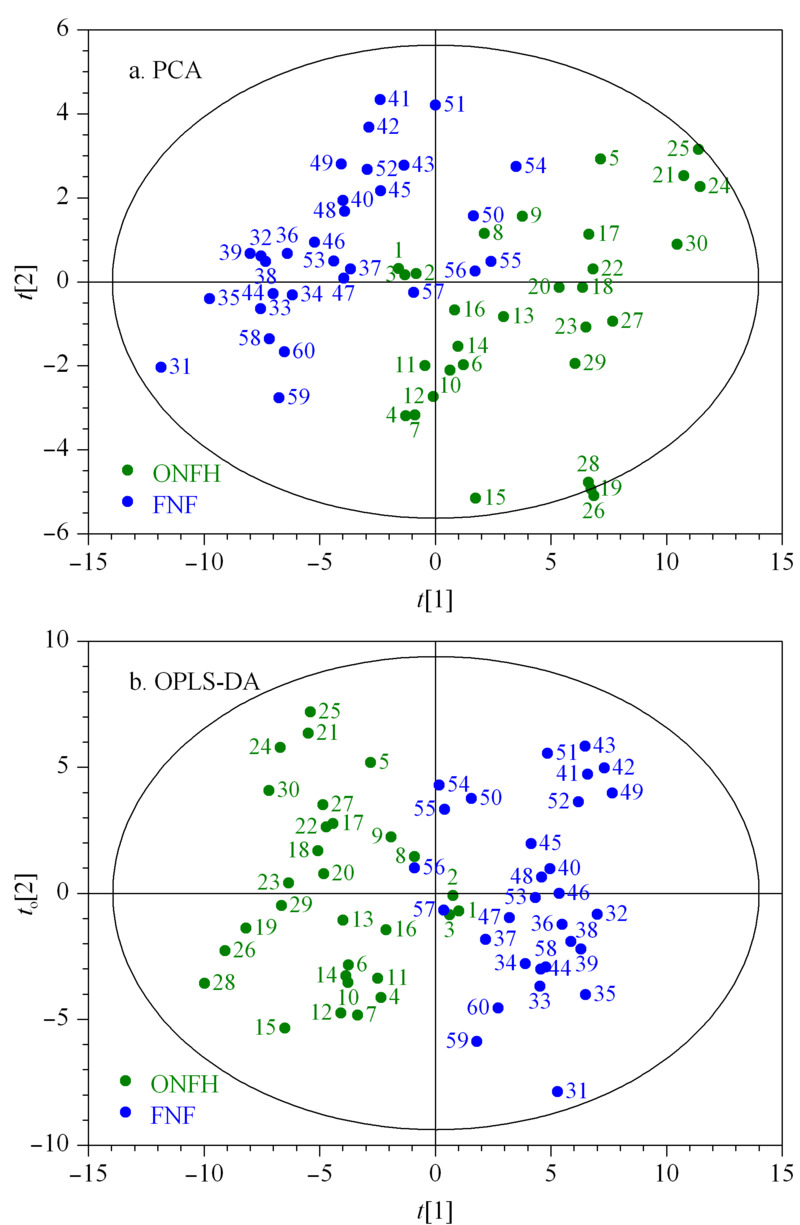
FNF和ONFH患者的外泌体样本的(a)PCA和(b)OPLS-DA评分图

模型评价参数*R*^2^*Y*、*Q*^2^分别为0.749、0.724, 均大于0.5,表明模型稳定可靠(见图3b)。表明OPLS-DA模型建立成功,来源于ONFH和FNF骨组织的外泌体分离趋势较好。

### 2.4 差异代谢物热图

根据VIP >1和变化倍数>2的标准,在外泌体的脂质代谢物中筛选了18个差异代谢物(见表2),包括丙烯醇脂类、脂肪酸酯类、甘油酯类及其衍生物。热图显示了表2中差异代谢物的变化:在ONFH中有12个脂质代谢物的表达增加,而其他6个脂质代谢物的表达减少(见图4)。

**表 2:** 外泌体差异代谢物列表

Metabolite	m/z (Da)	t_R_/s	VIP	P	HMDB ID	Chemical formula	Average M_r_
Phosphatidylserine	424.279	462.799	2.170	0.014	HMDB0014291	C_13_H_24_NO_10_P	385.304
1-(O_2_-(2-methylamino-2-oxo-ethyl)-O_5_-hydroxy-	432.262	460.255	3.215	0.008	HMDB0013031	C_9_H_13_N_5_O_3_	239.231
phosphinyl-β-d-ribofuranosyl) thymine							
LysoPE(18∶3(6Z,9Z,12Z)/0∶0)	440.256	461.135	1.192	0.000	HMDB0011508	C_23_H_42_NO_7_P	475.555
Lucyoside K	597.376	336.932	1.043	0.001	HMDB0041353	C_36_H_56_O_9_	632.824
Physapruin B	641.289	538.855	3.067	0.016	HMDB0040671	C_34_H_50_O_9_	602.755
Carfentrazone-ethyl	435.191	536.218	1.768	0.000	HMDB0041477	C_8_H_18_S	146.294
LysoPC(18∶2(9Z,12Z))	520.339	563.929	1.040	0.014	HMDB0010386	C_26_H_50_NO_7_P	519.651
1-Palmitoyl-Sn-Glycero-3-Phosphocholine	496.340	589.061	3.241	0.000	HMDB0010382	C_24_H_50_NO_7_P	495.630
LysoPC(20∶4(5Z,8Z,11Z,14Z))	544.339	605.876	1.518	0.001	HMDB0010395	C_28_H_50_NO_7_P	543.672
1-Oleoyl-Sn-Glycero-3-Phosphocholine	522.356	605.999	1.304	0.000	HMDB0002815	C_26_H_52_NO_7_P	521.667
(±)-Abscisic acid	265.151	359.008	1.277	0.023	HMDB0035140	C_15_H_20_O_4_	264.316
Methyl methylthio selenide	142.948	72.309	1.957	0.000	HMDB0030879	C_2_H_6_SSe	141.09
Pubescenol	492.321	365.979	1.648	0.000	HMDB0030085	C_28_H_42_O_6_	474.629
Nonacosan-10-one	423.788	372.576	3.079	0.000	HMDB0033719	C_29_H_58_O	422.770
(24E)-3α-Acetoxy-15α-hydroxy-23-oxo-7,9(11),	491.321	371.631	2.808	0.000	HMDB0035386	C_32_H_46_O_6_	526.704
24-lanostatrien-26-oic acid							
Ganoderic acid Mg	620.406	367.133	2.732	0.016	HMDB0035999	C_35_H_54_O_8_	602.798
PA (8∶0/12∶0)	498.310	368.099	1.056	0.005	HMDB0115483	C_23_H_45_O_8_P	480.579
Taurolithocholic acid	484.316	366.788	1.056	0.005	HMDB0000722	C_26_H_45_NO_5_S	483.71

VIP: variable importance in the projection value of OPLS-DA models. Differences were considered significant at *P*<0.05.

**图 4:**
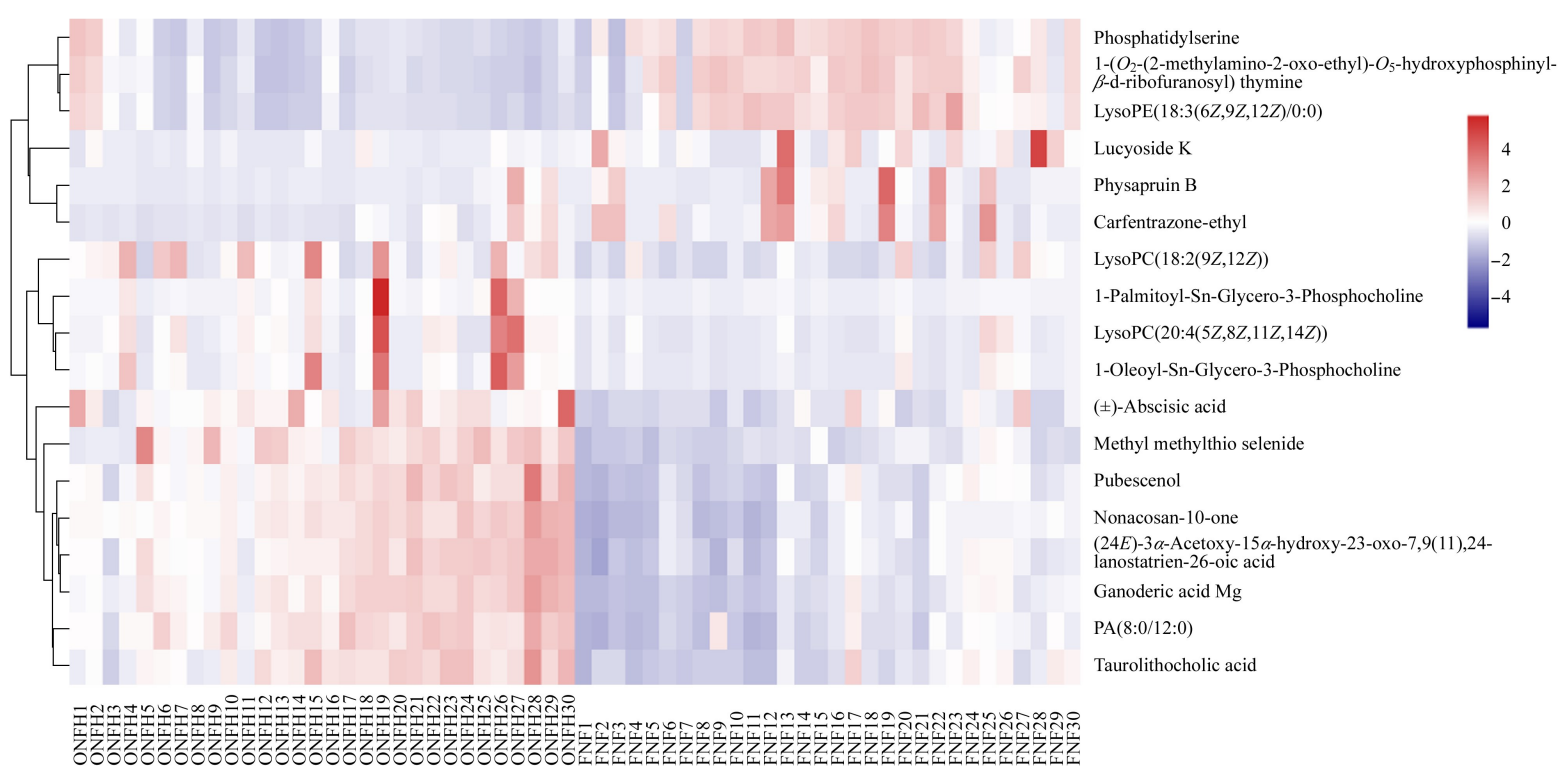
差异代谢物的热图

### 2.5 代谢途径分析

将脂质代谢物(见附表1,详见http://www.chrom-China.com)导入MetaboAnalyst网站(https://www.metaboanalyst.ca/)进行代谢通路分析。代谢通路分析显示,甘油磷脂代谢和鞘脂类代谢通路在来自于ONFH的外泌体中发生了较大的改变(见图5)。有研究表明,鞘脂和甘油磷脂之间的不平衡导致脂肪毒性损伤,这与常见代谢性疾病的病理生理学有关^[17]^。Han等^[18]^认为,甘油磷脂代谢途径对人类多能干细胞的多能性和生存的铁稳态至关重要。还有研究揭示了甘油磷脂与细胞增殖、分化和凋亡之间具有相关性,甘油磷脂比例的变化可以反映脂质代谢的紊乱^[19]^。Zhu等^[16]^的研究认为,在ONFH疾病中,脂质代谢紊乱是一个重要的病理因素。外泌体内的甘油磷脂代谢和鞘脂类代谢可能也代表了疾病本身的代谢变化。

**图5:**
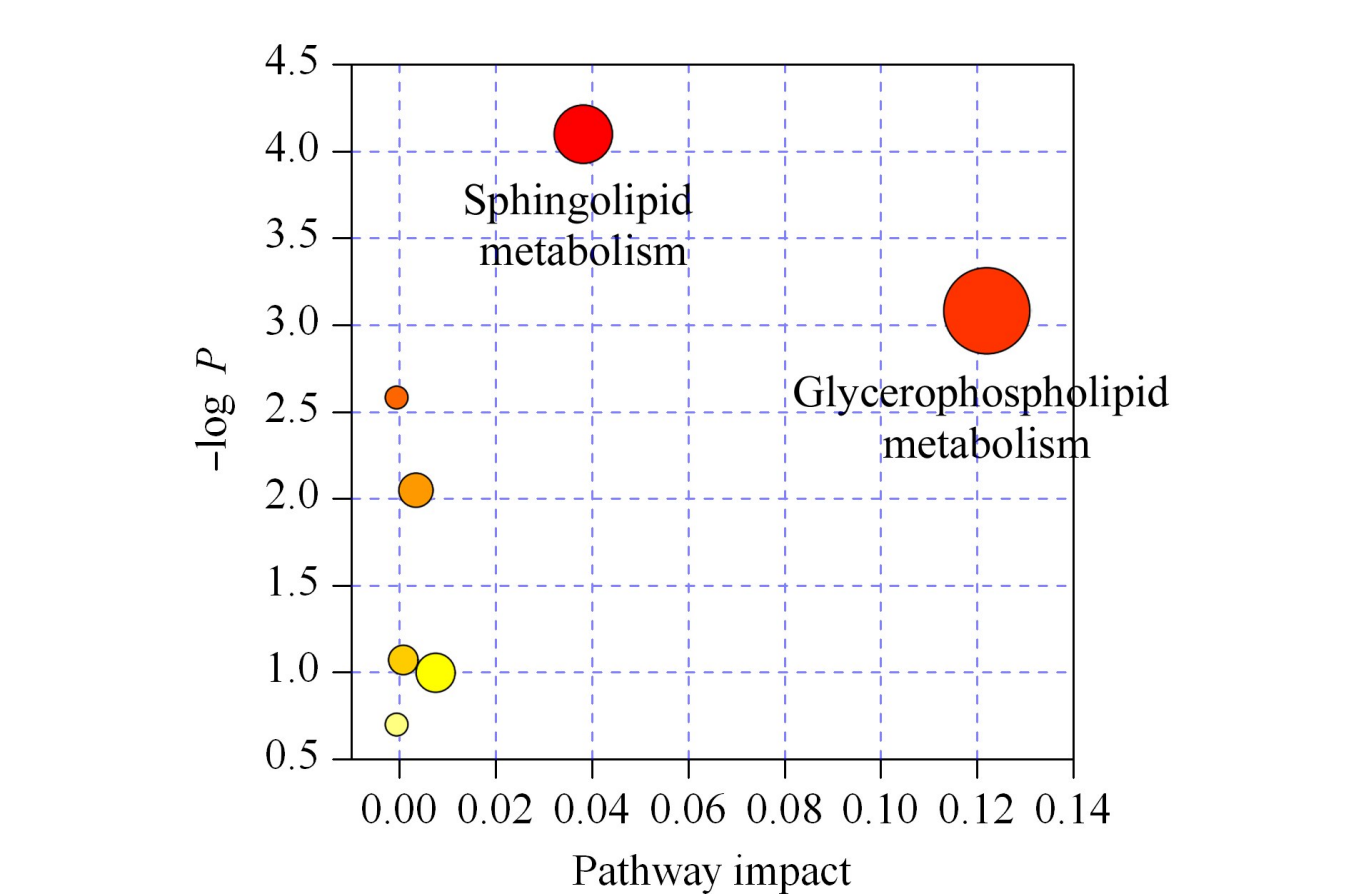
ONFH患者外泌体中已鉴定代谢物的代谢途径分析

## 3 结论

在本研究中,使用基于UPLC-MS/MS的非靶向代谢组学方法识别来源于ONFH和对照组股骨头组织的外泌体之间的脂质代谢差异,并发现了ONFH外泌体的代谢特征,共鉴定出18种差异代谢产物,包括丙烯醇脂类、脂肪酸酯类、甘油酯类及其衍生物。脂质代谢紊乱是ONFH的基本特征之一,外泌体脂质代谢组学可能是揭露ONFH疾病脂质代谢变化的一个重要方法。
